# Proteomics of Plasma and Plasma-Treated Podocytes: Application to Focal and Segmental Glomerulosclerosis

**DOI:** 10.3390/ijms241512124

**Published:** 2023-07-28

**Authors:** Cerina Chhuon, Luis Vicente Herrera-Marcos, Shao-Yu Zhang, Cécile Charrière-Bertrand, Vincent Jung, Joanna Lipecka, Berkan Savas, Nour Nasser, André Pawlak, Hocine Boulmerka, Vincent Audard, Dil Sahali, Ida Chiara Guerrera, Mario Ollero

**Affiliations:** 1 Proteomic Platform Necker, Université Paris Cité Structure Fédérative de Recherche SFR Necker US24, 75015 Paris, France; cerina.chhuon@inserm.fr (C.C.); vincent.jung@inserm.fr (V.J.); joanna.lipecka@inserm.fr (J.L.); 2 Univ Paris Est Creteil, INSERM, IMRB, F-94010 Creteil, France; luis.herrera-marcos@inserm.fr (L.V.H.-M.); shao-yu.zhang@inserm.fr (S.-Y.Z.); charriere@u-pec.fr (C.C.-B.); berkan.savas@inserm.fr (B.S.); nour.nasser@inserm.fr (N.N.); andre.pawlak@inserm.fr (A.P.); hocine.boulmerka@hotmail.fr (H.B.); vincent.audard@aphp.fr (V.A.); dil.sahali@inserm.fr (D.S.); 3 AP-HP, Hôpitaux Universitaires Henri Mondor, Service de Néphrologie, F-94010 Creteil, France

**Keywords:** podocytopathy, lipid rafts, raftomics, phosphoproteomics, extracellular vesicles, immunodepletion

## Abstract

Focal and segmental glomerulosclerosis (FSGS) is a severe form of idiopathic nephrotic syndrome (INS), a glomerulopathy of presumably immune origin that is attributed to extrarenal pathogenic circulating factors. The recurrence of FSGS (rFSGS) after transplant occurs in 30% to 50% of cases. The direct analysis of patient plasma proteome has scarcely been addressed to date, mainly due to the methodological difficulties associated with plasma complexity and dynamic range. In this study, first, we compared different methods of plasma preparation, second, we compared the plasma proteomes of rFSGS and controls using two preparation methods, and third, we analyzed the early proximal signaling events in podocytes subjected to patient plasma, through a combination of phosphoproteomics and lipid-raft proteomics (raftomics). By combining immunodepletion and high pH fractionation, we performed a differential proteomic analysis of soluble plasma proteins and of extracellular vesicles (EV) obtained from healthy controls, non-INS patient controls, and rFSGS patients (n = 4). In both the soluble- and the EV-protein sets from the rFSGS patients, we found a statistically significant increase in a cluster of proteins involved in neutrophil degranulation. A group of lipid-binding proteins, generally associated with lipoproteins, was found to be decreased in the soluble set from the rFSGS patients. In addition, three amino acid transporters involved in mTORC1 activation were found to be significantly increased in the EV from the rFSGS. Next, we incubated human podocytes for 30 min with 10% plasma from both groups of patients. The phosphoproteomics and raftomics of the podocytes revealed profound differences in the proteins involved in the mTOR pathway, in autophagy, and in cytoskeleton organization. We analyzed the correlation between the abundance of plasma and plasma-regulated podocyte proteins. The observed changes highlight some of the mechanisms involved in FSGS recurrence and could be used as specific early markers of circulating-factor activity in podocytes.

## 1. Introduction

Idiopathic nephrotic syndrome (INS) is a group of chronic diseases characterized by proteinuria, hypoalbuminemia, dyslipidemia, and edema [1]. Although the ultimate cause is currently unknown, a strong body of evidence suggests a link to immune dysfunction [2,3,4,5,6]. No specific treatment is available, but corticosteroids and immunosuppressants currently constitute the first-line therapies. Focal and segmental glomerulosclerosis (FSGS) is the most frequent form of INS among adults. The diagnosis of FSGS relies exclusively on kidney biopsy, a highly invasive procedure, due to the lack of diagnostic and prognostic biomarkers. The disease is progressive, can be unresponsive to therapy, and eventually leads to end-stage renal insufficiency requiring lifetime dialysis or kidney transplantation. However, 30% of transplanted individuals develop a recurrence of the disease (rFSGS) in the hours or days following renal graft [7]. Compelling evidence suggests the existence of a causative permeability factor responsible for rFSGS [8]. The latter is commonly managed by plasma exchange (PE) or plasmapheresis, a therapy in which plasma is removed and replaced by an albumin solution. Furthermore, PE is used for the removal of deleterious proteins or antibodies, and it is widely employed in auto-immune diseases, neurologic pathologies, hepatic insufficiency, and familial hypercholesterolemia [9]. This intervention has proven to be an effective treatment for rFSGS, though its benefit is usually limited to several months. Blood plasma represents both a unique source of information and a highly challenging material. Several groups have tried to identify the permeability factor or factors responsible for the disease’s recurrence through various methods, and several potential candidates have been suggested (for a review, see [10,11,12]). Only a limited number of studies have used untargeted mass spectrometry to identify novel candidates in plasma, following affinity chromatography [13] or two-dimensional electrophoresis [14].

In order to identify biomarkers or pathogenic factors in plasma, the in-depth characterization of low- and medium-abundance proteins is mandatory. The depletion of high-abundance proteins has been used to closely examine the plasma proteome [15,16]. More recently, a semi-comprehensive analysis of the circulating proteome was conducted using aptamer-based capture strategies [17]. A growing number of studies have focused on extracellular vesicles (EV). These EV have been found to be involved in the pathogenesis of kidney diseases [18,19] and play important roles in immune regulation, including the activation and inhibition of immune-cell responses [20]. They provide a snapshot of the cell from which they originate at a particular moment, which makes them invaluable tools for the diagnosis, prognosis, and progression assessment of diseases. To date, a few studies have analyzed EVs in PE samples, in the search for the miRNA [21] and proteomic signatures [22] of patients.

In contrast to the direct analysis of circulating materials, the study of podocyte-signaling events in response to recurrent INS plasma/serum has unveiled or confirmed the involvement of several pathways and proteins in rFSGS, such as the TNF-α [23] and β3-integrin [24,25] pathways, proteins of the slit diaphragm, like podocin and nephrin [26], TRPC channels [27], protease-activated receptor 1 (PAR1) activity, vasodilator-stimulated phosphoprotein (VASP) phosphorylation [28], the small GTPase Rac1 [29], cytoskeletal proteins [30], and lipid-metabolism-related proteins [31]. Many of these components are present in or associated with raft-like membrane microdomains, suggesting that the dynamics of the raft recruitment of proteins can trigger intracellular signaling responses. This implies that early proximal events are decisive in the fate of podocyte structures, and therefore, of glomerular function. Global-molecular-analysis approaches have never been used in this context. 

In the present work, we compared different methods of sample preparation and analysis to compare the protein composition of EVs and depleted PE proteins between rFSGS and control patients in order to identify novel noninvasive biomarkers of the disease. In addition, we analyzed the responses of human podocytes to short contact—30 min—with plasma-exchange fluids from rFSGS patients. Finally, we correlated the plasma analyses with those of the raft proteome and total cell phosphoproteome.

## 2. Results

### 2.1. Clinical Characteristics of Patients

In this study, we aimed, first, to develop the analytical conditions to explore the first PE proteome of rFSGS patients in order to identify low-abundance and potentially clinically discriminating proteins. Second, we sought to characterize the changes in podocyte-signaling networks in response to short exposure to the first PE fluid obtained from four rFSGS patients and four non-INS patients undergoing immunological graft rejection. The latter were used as controls. All the rFSGS patients developed proteinuria immediately or within days after surgery. To prevent allograft rejection, the patients were treated with corticosteroids and immunosuppressive agents. As drugs are known to induce profound changes in protein expression, we selected control patients who underwent renal transplantation and equivalent treatments to those of rFSGS patients, which included immunosuppressants, corticosteroids, PE, rituximab, and immunogloblin. The clinical characteristics are summarized in Table 1. The first PE samples were drawn from the patients, immediately frozen, and stored at −80 °C until use.

### 2.2. Comparison of Blood-Plasma and Plasma-Exchange-Fluid Proteomes 

For the soluble-PE-protein identification, we chose to build a spectral library through a combination of immunodepletion—allowing us to deplete the 12 most abundant proteins in the plasma (TOP12)—and extensive high-pH reverse-phase-peptide fractionation (13 fractions, up to 26 h of machine time per sample). The library allowed us to transfer peptide identifications from one sample to another, without the need to fractionate every sample, which is time-consuming. The in-house plasma library contained 917 plasma proteins. For comparison, immunodepletion alone allowed the identification of 358 proteins, while extensive fractionation alone led to the identification of 406 proteins. Using the spectral library, we identified a higher number of proteins in these samples using a 2-h LC MSMS analysis (Figure S1). 

Samples of PE are readily available and stored in large quantities. However, the question is raised as to whether the PE proteome can be compared to that of peripheral blood plasma (e.g., from healthy donors, not subjected to PE). Considering the manner in which the plasmapheresis device operates, there is no clear evidence of any compositional differences between plasma and PE samples. Therefore, we aimed to compare the plasma and PE proteomes.

**Figure 1:**
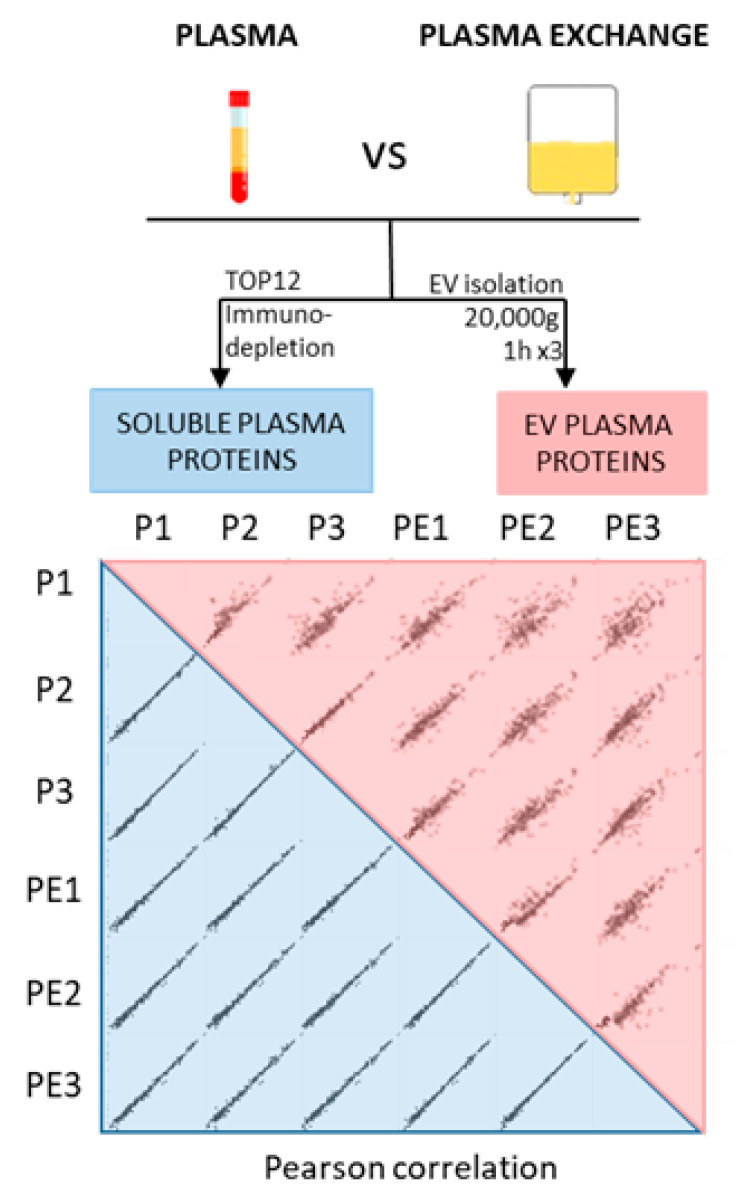
Comparison of PE and plasma samples. Plasma and PE were collected on the same day from a patient treated with plasmapheresis (non-recurrent FSGS facing humoral rejection). After collection, 500 µL (for EV preparation) and 10 µL (for soluble proteins preparation) of each sample were placed in 3 different tubes to generate separate sample triplicates for the analysis. Triplicates of plasma (P1, P2, and P3) and plasma exchange (PE1, PE2, and PE3) were analyzed to assess reproducibility of the preparation. Soluble (in blue) and EV (in red) plasma proteins were performed on both types of sample. Pearson correlation coefficients were calculated for the LFQ intensities of the triplicates to assess the similarity between plasma and PE.

To enlarge the spectrum of proteins analyzed within the plasma of the patients, we additionally analyzed the proteins contained in the plasma EVs. By exploring the soluble plasma proteome and the EV proteome, we covered two major fractions of plasmatic proteins. We performed immunodepletion and EV isolation on the plasma drawn from a patient who received PE therapy on the same day. The PE was collected immediately after the first plasmapheresis session. Each sample was divided into three parts, which were analyzed in parallel. The number of identified proteins was comparable between the plasma and PE samples for the soluble proteins (after immunodepletion), although we identified slightly lower EV proteins from the PE (Figure 1). 

To evaluate the similarity between the plasma and PE proteomes, we calculated the Pearson correlation coefficients of the LFQ (label-free quantitation) intensities for the soluble and EV proteins. The comparison of the soluble proteins showed a strong correlation (r = 0.995–0.998) (Figure S2, blue shades). The EV plasma proteins showed a slightly weaker correlation (0.77–0.942) between the plasma EV and the PE EVs (Figure S2, pink shades). Nonetheless, a certain variability was also observed between the vesicles prepared independently from the same sample, suggesting that EV preparation per se may have been the reason for the observed variability. The average Pearson correlation coefficients of the LFQ intensities for each group were >0.8 for the soluble proteins and EV proteins. Furthermore, to confirm the similarity between the proteomes, we plotted logarithmized the LFQ intensities of alpha-2-macrobulin (A2M) and serotransferrin (TF), both soluble plasma proteins, showing no significant differences (Figure 1). The same results were found for the EV-marker proteins, such as CD9 and integrins, which were also not significantly different.

### 2.3. Analysis of Soluble and EV Plasma Proteomes from rFSGS Patients

**Figure 2:**
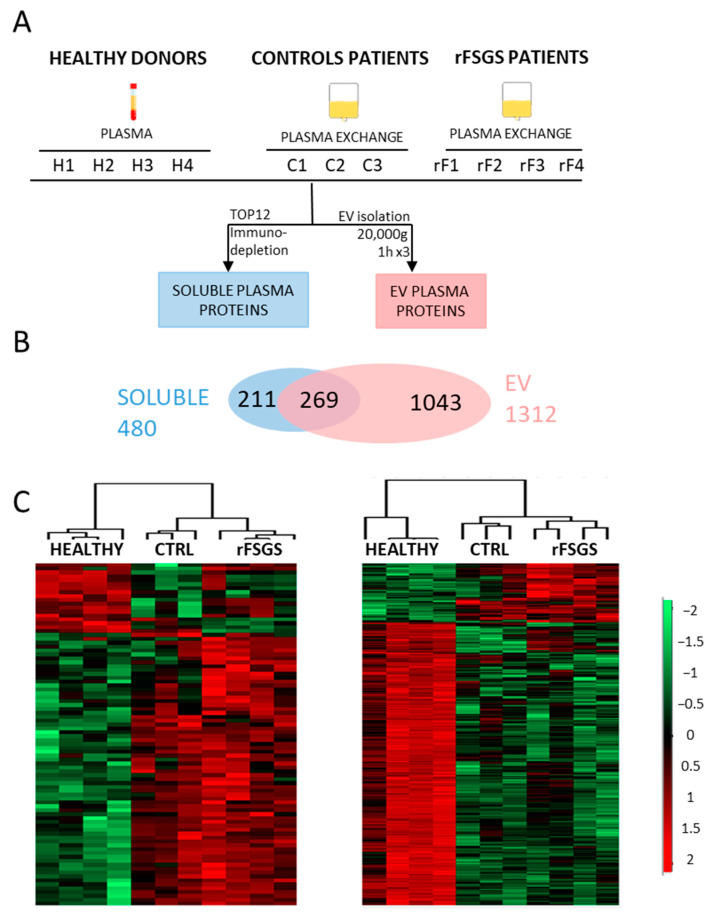
Comparison of plasma and EV proteomes from healthy individuals, control patients, and rFSGS patients. (**A**) Study workflow and design. For this study, plasma of four healthy donors (H1, H2, H3, and H4) and plasma exchange of 3 control patients (C1, C2, and C3, with humoral rejection post-transplantation) and 4 rFSGS patients (rF1, rF2, rF3, and rF4, with recurrent FSGS post-transplantation) were obtained to perform TOP12 depletion for the analysis of soluble plasma proteins (in blue) and EV isolation for the analysis of microvesicle plasma proteins (in pink). (**B**) Venn diagram representing the overlap between identified EV proteins and soluble plasma proteins. (**C**) Heatmap representation, using Perseus software (version 1.5.5.3), of significantly different proteins (ANOVA test, s0 = 0.1, Permutation-based FDR < 0.05) between rFSGS patients, healthy donors and control patients. The LFQ intensities of each protein (rows) were ranked and grouped according to their distribution signals in the clustering heatmap. Each column represents samples from each group. The color scale denotes abundance variation (red for more abundant, green for less abundant).

We collected the PE from the rFSGS patients and compared them with two sets of controls to find potential specific disease-associated proteins. These two sets of controls included peripheral blood plasma (from healthy donors) and PE (from CTRL patients). As no significant differences between the plasma and PE proteome profiles were found in the preceding study, we compared the proteins from the plasma of healthy donors and the PE from the rFSGS and control patients (Figure 2A). A total of 480 soluble plasma proteins and 1312 EV proteins were identified across all the samples, with 269 proteins in common (Figure 2B). Interestingly, we found Es markers of several immune cells in the EV (CD45 for leucocytes, CD8/81/82 for T-cells, and CD19/29/37 for B-cells) that we did not identify among the soluble proteins. The changes in plasmatic protein abundance are depicted in the heatmaps in Figure 2C. An ANOVA test (permutation-based FDR < 0,05) led to the quantification of 88 significantly different proteins among the three groups for the plasma-soluble proteins and 478 significantly modulated proteins for the EVs (Table S1). In both cases, the hierarchical clustering of the samples indicated that the significant proteins clearly discriminated the healthy donors, control patients, and rFSGS patients. The two groups of patients showed more similar profiles compared to the healthy controls.

In order to select disease-related proteins, we chose those differentially abundant in the rFSGS patients compared to the healthy donors and to the control patients. 

**Figure 3:**
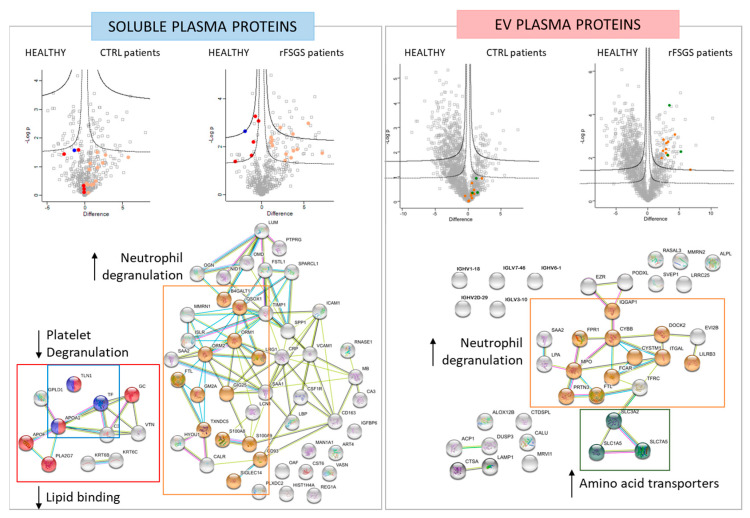
Differentially abundant proteins in soluble and EV experiments. For soluble and EV-plasma proteins, two volcano plots are depicted, and represents the log2 fold change (difference, *x*-axis) against the –log10 *p*-value (*y*-axis) for proteins differentially expressed between control–PE (right of plot) and healthy plasma (left of plot), and between rFSGS–PE (right of plot) and healthy plasma (left of plot). STRING network representation (bottom) of significantly different protein interactions. Proteins linked to neutrophil degranulation (in orange), platelet degranulation (in blue), lipid binding (in red), podocyte cytoskeleton (in purple), and amino acid transporters (in green) ae depicted in the STRING analysis.

Among the soluble proteins, 58 were modulated (47 upregulated and 11 downregulated) in the rFSGS patients relative to the two sets of controls. These proteins were subjected to a functional enrichment analysis using String-DB. Among the 47 upregulated proteins, 13 (B4GALT1, QSOX1, ORM1, ORM2, LRG1, FTL, GM2A, GIG25, TXNDC5, S100A8, S100A9, CD93, and SIGLEC14) are involved in neutrophil degranulation (FDR = 1.17 × 10^−8^) (Figure 3). Among the eleven downregulated proteins, three (TLN1, TF, and APOA1) are implicated in platelet degranulation (FDR = 0.0077), and five (TLN1, APOA1, GC, APOF and PLA2G7) are lipid-binding (FDR = 0.0018) (Figure 3). All these proteins are highlighted in the volcano plots, respecting the same color code.

Of the EV proteins, thirty-eight were modulated (thirty upregulated and eight downregulated) in the rFSGS patients compared to the two groups of controls. Of these, 11 (PRTN3, MPO, FTL, FPR1, CYBB, IQGAP1, CYSTM1, FCAR, ITGAL, DOCK2, LILRB3) are involved in neutrophil degranulation (FDR = 8.36 × 10^−9^). Therefore, this functional class was significatively enriched in rFSGS in both the soluble and the EV fractions. Interestingly, none of these proteins, except for the FTL, appeared in both sets, confirming the complementarity of the protein classes detected in the plasma and in the EVs. Another class that was specifically enriched and increased was that of the amino acid transporters, composed of the SLC7A5/SLC3A2 heterodimer, along with SLC1A5, which were previously described as involved in mTORC1 activation through nutrient sensing (18). For both the soluble and the EV proteomes, the groups of significant proteins were also represented in volcano plots (FDR < 0.05, s0 = 0.1, 250 randomizations), respecting the same color code as the functional annotations (Figure 3).

### 2.4. Raftomic Analysis of Podocytes Incubated with rFSGS and Control PE

To characterize the changes in the podocyte-signaling networks in response to the short exposure to patient PE (30 min), we first analyzed the lipid rafts using a proteomic approach we recently developed [32]. We identified, on average, 2626 and 2601 proteins in the raft fraction (3163 proteins in total) from the podocytes treated with the rFSGS and the control PE, respectively. The purity of the raft preparation was verified by Western blot for all the fractions from the Optiprep density gradient. We performed the immunostaining of flotilin-1, a protein-raft marker [33], and of β-actin, a cytosolic protein, as a non-raft marker. As shown in Figure S3A, a strong flotillin-1 signal and a low actin signal were detected in fraction 2, suggesting lipid-raft enrichment. This result was confirmed by the MS analysis (Figure S3B), as the intensities of both the flotillin-1 and the flotillin-2 were 23-fold higher in the raft preparation than in the total proteome. Finally, a Gene Ontology cellular-component analysis was performed for all 3163 raft proteins (Figure S3C). As expected, we observed a high number of membrane (2139/3163) and plasma-membrane proteins (703/3163). We also detected a high number of extracellular vesicular exosomes (1094/3163), which was also expected, as exosomes are associated with lipid rafts [34].

**Figure 4:**
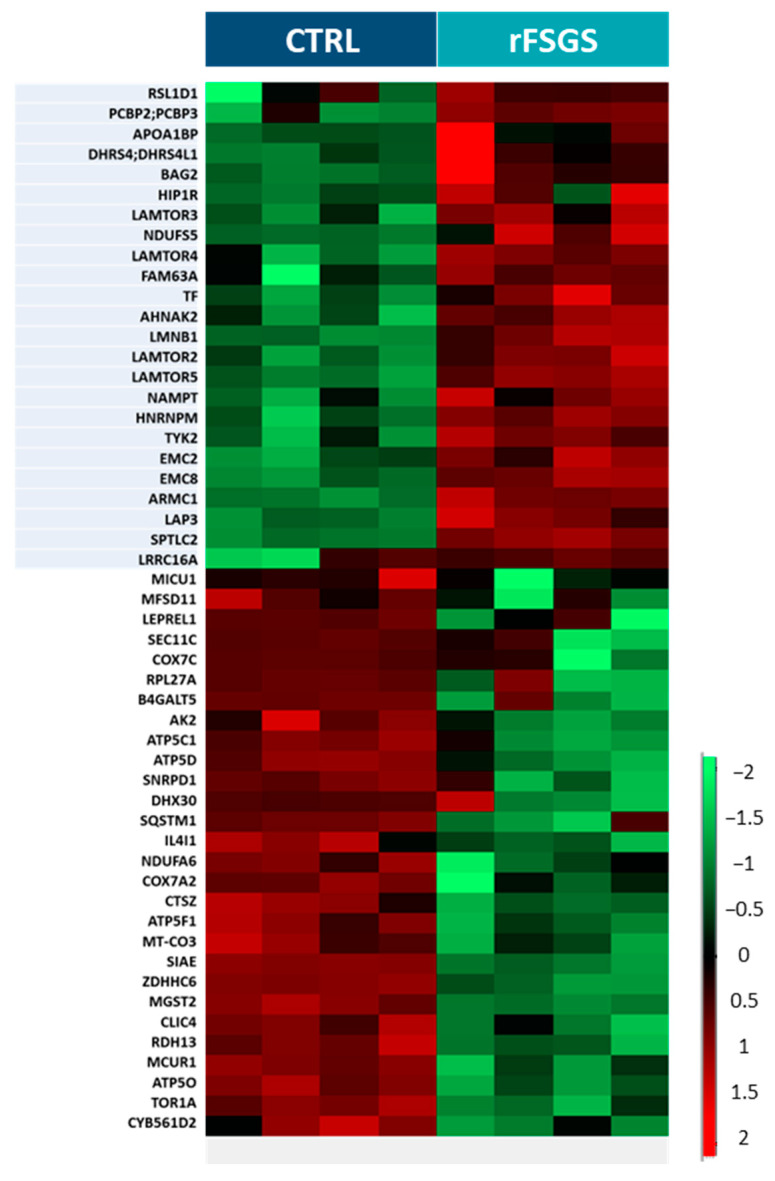
Proteins differentially recruited to lipid rafts. Heatmap of fifty-two proteins whose recruitment profiles were significantly different between control-PE- and rFSGS-PE-incubated podocytes. Each column represents a biological replicate (n = 4 for CTRL and n = 4 for rFSGS). Protein signals (rows) were ranked and grouped according to their distribution signals in the clustering heatmap. The color scale shown on the right side denotes the relative recruitment level of lipid rafts across all samples. Twenty-eight proteins with significantly decreased recruitment in lipid rafts (*t*-test, permutation-based FDR < 0.05) are shown in green, while twenty-four proteins with significantly increased recruitment in lipid rafts (*t*-test, permutation-based FDR < 0.05) are noted in red.

The changes in protein recruitment into lipid rafts after stimulation with rFSGS compared with the control PE are depicted in the heatmap in Figure 4. A total of four biological replicates for each condition were analyzed. The statistical analysis (Student’s *t*-test with permutation-based FDR < 0.05) of the lipid rafts led to the identification of 52 significantly modulated proteins in response to PE, which were separated into two major clusters by hierarchical clustering. Twenty-four proteins were more heavily recruited into the rafts, and twenty-eight were less heavily recruited into the rafts following the rFSGS PE incubation compared to the control PE incubation (Table S2). Interestingly, four proteins (LAMTOR-2, LAMTOR-3, LAMTOR-4, and LAMTOR-5) in the top cluster belonged to the ragulator complex, while seven proteins in the bottom cluster (ATP5O, ATP5D, ATP5F1, ATP6C1, MT-CO3, COX7A2, and COX7C) are involved in mitochondrial function.

### 2.5. Phosphoproteomic Analysis of Podocytes Incubated with rFSFG and Control PE

To further investigate the early cellular pathways affected by the patients’ plasma components, we carried out a phosphoproteomic analysis upon the 30-min PE treatment. The podocytes were processed separately for the analysis of the total proteome and phosphoproteome. The phosphopeptide enrichment with TiO_2_ tips was performed on four biological replicates for each group. As expected from this method, the majority of the phosphoproteins were singly phosphorylated, with only 22% of the identified phosphoproteins presenting more than one phosphosite (Figure S4A). We achieved a high-specificity enrichment, as 91% of all the identified peptides were phosphopeptides (Figure S4B). Phosphorylation on the Ser residues was predominantly identified (92.6%), followed by phosphorylation on Thr (7.1%) and Tyr (0.3%), as shown in Figure S4C. A total of 7884 phosphosites were identified at least once from a total of 8688 peptides. On average, we identified 4870 and 4900 phosphosites (5084 phosphosites in total) in the podocytes treated with the rFSGS and the control PE, respectively, with a minimum of four values in at least one group and a phosphosite-localization probability of over 75%, as shown in Figure S4D (corresponding to the quantification of 1972 and 1990 phosphoproteins). When we applied the Student’s *t*-test (permutation-based FDR < 0.05), we found 66 phosphorylation sites to be significantly modulated following the rFSGS incubation: 32 phosphosites were upregulated and 34 phosphosites were downregulated. These differentially displayed phosphosites are shown in Table S3. Among these phosphoproteins, thirteen are associated with cytoskeleton rearrangement (MPRIP, OSBPL3, HSPB1, PLEC, LARP1, AKAP12, MAP7D1, LMNA, NUFIP1, SIPA1L2, CLIP2, NCK2, and SASH1) and five are involved in mTOR activation (EIF4ENIF1, NUFIP1, BRD4, LARP1, and SLC12A7).

### 2.6. Data Integration of Proteomic Analyses on Podocytes

**Figure 5:**
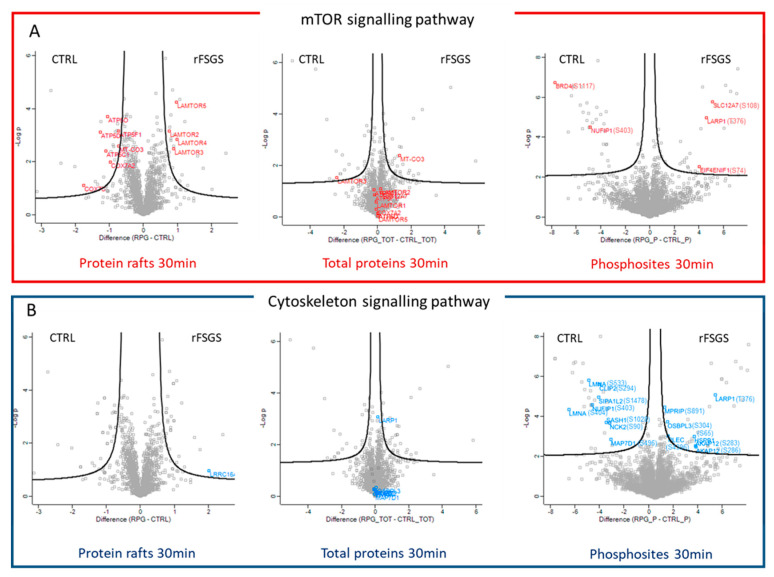
Differentially expressed proteins in response to rFSGS PE are mostly involved in mTOR activation and cytoskeleton rearrangement. Volcano plots representing the log2 fold change (difference, *x*-axis) against the –log10 *p*-value (*y*-axis) for proteins differentially expressed or recruited between control–PE (left of plot)- and rFSGS–PE (right of plot)-incubated podocytes. Raft proteome (left plot), total proteome (center plot), and phosphoproteome (right plot) at 30 min are represented. (**A**) Proteins linked to mTOR activation are labeled with red. (**B**) Proteins linked to cytoskeleton regulation are labeled with blue.

The results obtained by our two complementary proteomic analyses were integrated into multiple volcano plots (Figure 5). The proteins involved in the mTOR signaling pathway are labeled with red (Figure 5A) and the cytoskeleton-associated proteins are labeled with blue (Figure 5B) for the three different analyses performed (raft proteome on left panels, total proteome on center panels, and phosphoproteome on right panels). The total proteome was analyzed in parallel to verify that the changes in the phosphorylation levels or in the raft recruitment were not due to changes in the total cell-protein levels after the PE stimulation. Of the phosphoproteins involved in cytoskeleton rearrangement, eight were also quantified in the total proteome analysis, while five were not detectable. No significant expression changes were observed in the total cell extracts, confirming the actual change in the phosphorylation of these proteins. Furthermore, the proteins that were differentially recruited in the rafts were not differentially expressed in the total cell extracts, except for a slight difference in MT-CO3 and LAMTOR 3, but in the opposite sense to the rafts. Altogether, the total-cell-expression data support the hypothesis that the observed changes corresponded to differences in raft recruitment. Among the differential phosphoproteins involved in cytoskeleton regulation, only LRRC16A, a protein that plays a role in the regulation of actin polymerization [35], was found to be more heavily recruited in the rafts after the rFSGS PE incubation. The integration of the raft-proteome and phosphoproteome analyses showed that the proteins participating in mTOR activation were modulated in both compartments. The raft-proteome analysis showed that the presence in the rafts of the ragulator complex and some mitochondrial proteins was induced by rFSGS PE, while five phosphosites belonging to five phosphorylated proteins involved in the mTOR pathway were upregulated by the effect of the rFSGS PE.

### 2.7. Targeted Analysis of Selected Proteins

**Figure 6:**
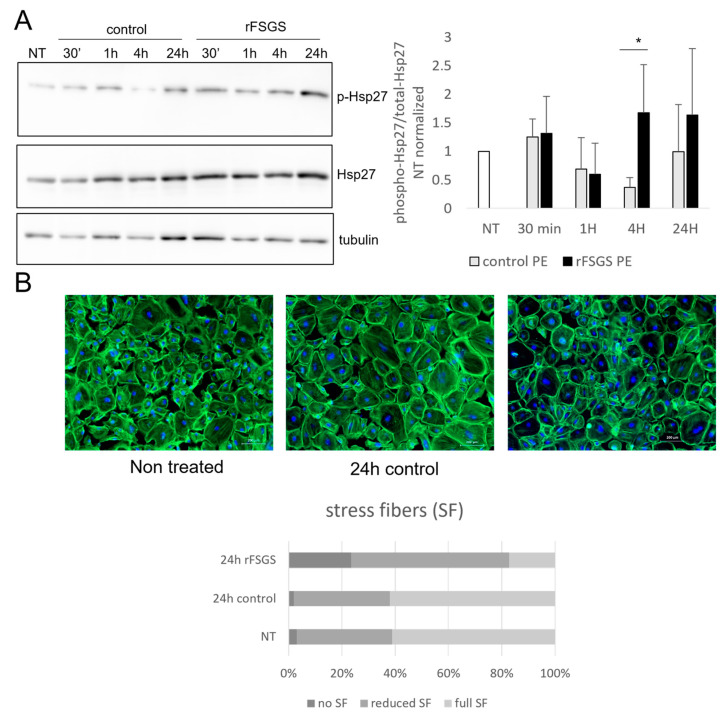
PE’s effect on targeted protein expression and podocyte morphology. (**A**) Human podocytes were subjected to 4 h starvation, and then incubated for 30 min, 1 h, 4 h, or 24 h in the presence of 10% rFSGS or control PE fluid. Non-treated cells (NT) were used as controls. Total protein extracts were subjected to SDS-page electrophoresis and Western blot, using specific antibodies to phosphorylated (Ser82) and total Hsp27. The bar diagram shows the phospho-Hsp27/total Hsp27 ratio after quantification of bands in three independent experiments (*: *p* < 0.05). (**B**) Fluorescence-microscope images of podocytes stained with FITC-tagged phalloidin, which labels actin fibers. Cells were either non-treated (NT) or incubated for 24 h in the presence of 10% control PE or rFSGS PE. Scale bar: 200 µm. Lower panel: quantification of stress fibers (SF) in podocytes. Three groups of cells are considered,: those with full length SF, those with reduced numbers or lengths of SF, and those with no CF. At least 3 treatments and 200 cells were counted per condition.

Most of the differentially expressed phosphosites identified in our work have only been described in previous phosphoproteomic studies, and no specific detection antibodies are currently available. This is the case for Hsp27, which we chose from within this group for further analysis, since we found significantly increased phosphorylation of the Ser65 site at 30 min of the rFSGS PE incubation. Since there were no available antibodies that recognized Ser65, we analyzed instead the time-course evolution of the Ser82 phosphorylation, and the results are shown on Figure 6A. In this series of experiments, the podocytes were incubated with PE for 30 min and for longer periods (1 h, 4 h, and 24 h), in a time-course fashion, and the total cell-protein extracts were obtained. We used the non-treated cells as additional controls. We postulated that early signaling changes after 30 min of incubation would result in effector-protein changes after longer exposure. Remarkably, after 4 h of incubation, the phosphorylation levels differed significantly between the rFSGS- and the control-PE-treated cells; the former increased and the latter decreased compared with the non-treated cells. This difference was maintained after 24 h, but it was not statistically significant. Since Hsp27 is involved in cytoskeleton regulation, we evaluated the morphology of the F-actin fibers through phalloidin staining and fluorescence microscopy. As shown in Figure 6B, the rFSGS–PE incubation resulted in stress-fiber loss after 4 h of incubation. 

### 2.8. Correlation of Plasma Proteomics and Podocyte Proteomics

**Figure 7:**
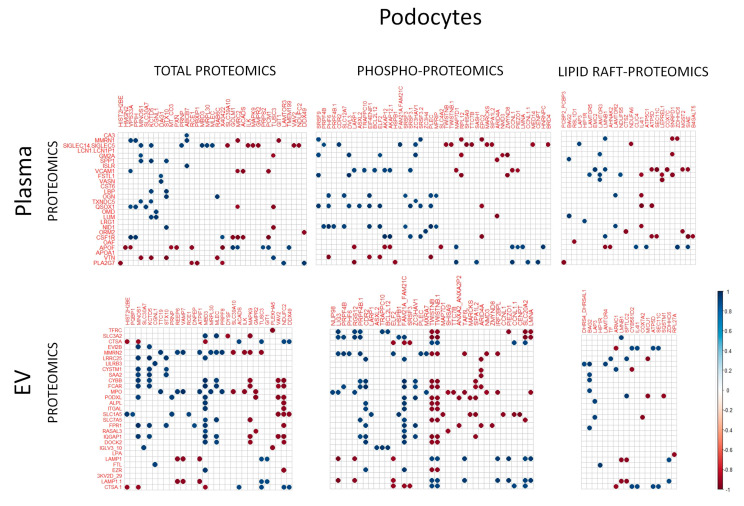
Correlation between PE proteomics and PE-treated podocyte proteomics. Spearman correlations were calculated between proteins found to be differentially present in PE (either soluble or EV fraction) and proteins found to be differentially expressed (total proteomics), phosphorylated (phosphoproteomics), or raft-localized (lipid raft proteomics) in podocytes incubated for 30 min with the same PE samples. The results are presented as correlation matrices, in which correlations with blue spots correspond to positive correlations and red spots correspond to negative correlations; r > 0.9 or r < −0.9 are considered.

Finally, we aimed to establish correlations between the proteins found in the plasma and the proteins that were changed in the plasma-treated podocytes. To this end, we limited the study to the proteins found to be significantly different between the rFSGS and the control conditions. The raw abundance of the proteins in each plasma dataset (soluble or EV) was correlated (Spearman correlation) with that in each podocyte dataset (total, raft, and phosphoprotein) (n = 7). In total, 2 × 3 correlation sets were established. Only “r” values over 0.9 or below −0.9 were considered, and they are shown in Figure 7; the positive correlations are in blue and the negative correlations are in red. Only the proteins highly correlated with at least one other protein are shown. Generally, the EV proteins showed more strong correlations with the podocyte proteins than with the plasma-soluble proteins. Among the plasma proteins showing the highest numbers of correlations were SIGLEC5/14 (a family of sialic-acid-binding proteins), VCAM 1, and apolipoprotein F (APOF) in the soluble fraction, as well as Multimerin 2 (MMR2) and myeloperoxidase (MPO) in the EV. Among the podocyte proteins, MBD3 and NDUFC2 in the total cell fraction and FAM21 and TWISTNB in the phosphoprotein fraction presented higher numbers of correlations with the plasma proteins. Interestingly, the differential raft proteins showed fewer correlations with both the plasma and the EV proteins.

## 3. Discussion

The present work addresses, on one hand, the proteomic analysis of plasma-exchange fluid from INS patients experiencing post-transplant FSGS recurrence (rFSGS) and, on the other hand, the search for early proteome changes in a cell target, in this case, podocytes, in response to rFSGS plasma.

### 3.1. Plasma-Proteome Analysis

Large amounts of plasma are often required in research aiming at the purification of low-abundance molecules for the diagnosis, prediction, and progression-tracking of a disease, such as circulating DNA [36], mRNA [37], miRNA [38], and proteins [39]. The plasma availability and the yield of bioactive molecules represent serious limitations on quantity and quality in clinical applications. The plasma proteome has a large dynamic range [40], with the concentration of high- and low-abundance proteins spanning 12 orders of magnitude, and the 10 most abundant proteins representing 90% of the total protein mass. High-abundance proteins are well characterized and understood, while low-abundance proteins are thought to be of great interest for their biological relevance and functions [41]. Immunodepletion using antibodies raised against the most abundant proteins is the most common method to identify low-abundance proteins. Other methods for plasma depletion include dye–ligand, protein–ligand, precipitation, the use of a combinatorial peptide–ligand library, and the enrichment of a protein population of interest, like EVs [16]. 

Extracellular vesicles (EVs), comprising microvesicles and exosomes, are released by most of the cells in human bodily fluids. They act as cellular messengers in cell-to-cell communication. Their composition has been found to differ in various pathologies and to be modulated by different stress conditions (chronic inflammation, infection, hypoxia, oxidative and mechanical stress, etc.) [42]. The EVs in blood mostly originate in platelets, endothelial cells, erythrocytes, and leukocytes, but they also arise in other cells in the body. Technical challenges have been encountered in the isolation of EVs from urine [43], sweat [44], cerebrospinal fluid [45], and bronchoalveolar lavage [46]. Arguably, the isolation and purity of EVs are even more difficult to achieve in blood because of the physicochemical properties of this fluid (i.e., the viscosity and the high abundance of some proteins). However, although plasma-EV isolation is challenging, its analysis represents an alternative in the search for plasma protein markers. Nevertheless, soluble and EV proteins may have different cellular origins and represent distinct pathways; consequently, their analysis is complementary. 

Compelling evidence suggests that one or several permeability factors are responsible for the pathological process leading to FSGS recurrence in some transplanted patients. To date, a limited number of proteomic analyses of serum/plasma in the context of FSGS have been conducted [13,14]. In the present work, we evaluated the differences in protein content between peripheral blood plasma and PE fluid from the same individual. This part of the study was particularly important, in that our results justified the comparison of both types of sample, which arguably correspond to the same biological material. Next, we implemented a method for EV proteomics and performed, in the best possible conditions, the differential analysis of rFSGS material in parallel with those from two types of control, namely, healthy individuals and control patients. The latter were non-FSGS patients who had been subjected to kidney transplant and, consequently, had received the same immunosuppressant therapy. 

In the best analytical setting that we could establish, we consistently found, within both the soluble proteins and the EV-associated proteins, an enrichment of rFSGS-upregulated proteins associated with neutrophil degranulation, in which FTL (ferritin light chain) appeared to be increased in both the soluble and the EV-protein sets. A neutrophil protein and granule component (gelatinase-associated lipocalin, NGAL) appeared in a urine-proteomic study as a biomarker of FSGS compared to MCNS, another form of INS, and its expression was correlated with the degrees of tubulointerstitial lesions [47], confirming the findings of previous reports [48,49,50,51], in which NGAL was presented as a reliable biomarker to distinguish both forms of INS. In line with our findings, FSGS has been reported in patients with severe neutrophilic leukemia [52]. Interestingly, we found NGAL in both the soluble and the EV set. While in the soluble set, the NGAL was increased in both the rFSGS and control patients compared to the healthy controls, in the EV, the increase was significant and specific in the rFSGS samples. Our results support the previous reports of NGAL as an FSGS marker, along with the hypothesis and observations of increased neutrophil activation in FSGS.

Conversely, a set of three proteins involved in platelet degranulation were downregulated in the rFSGS in the soluble plasma protein samples. Little is known about platelet activity in FSGS. In a comparative study of different INS forms, FSGS and MCNS patients showed decreased platelet reactivity compared to idiopathic membranous nephropathy [53]. Higher platelet counts have been reported in children with FSGS than in children with steroid-sensitive nephrotic syndrome [54]. Thromboembolism has been reported in some patients with INS [55], as has essential thrombocythemia [56]. 

Although the alteration in platelet activation in the rFSGS cannot be clearly inferred from our results, two of these three proteins (TLN1 and APOA1) were included in another cluster, described as lipid-binding, along with GC, APOF, and PLA2G7. Of these, APOA1, APOF, and PLA2G7 are components of lipoproteins. Indeed, dyslipidemia is one of the hallmarks of idiopathic nephrotic syndrome [57]. Remarkably, LDL apheresis has been proposed and tested as an alternative therapeutic approach in FSGS [58,59,60], and some genetic variants in apolipoprotein genes, namely APOE5 [61] and APOL1 [62], have been found to be associated with FSGS susceptibility. Familial hypercholesterolemia has also been described [63]. It would therefore be expected to find changes in lipoprotein-associated proteins in rFSGS-soluble plasma samples. The specific roles in FSGS of APOA1, APOF, and PLA2G7, and the relevance of their decreased presence, remain to be established.

A final major observation in our study was the increased presence of amino acid transporters in the rFSGS EV. The SLC7A5/SLC3A2 couple, which is known to heterodimerize, maybe a link between FSGS and mTOR signaling. In fact, these transporters were previously described as involved in the activation of mTORC1 through nutrient sensing [64]. The inhibition of mTOR signaling [65], specifically of mTORC1 [66], has been shown to decrease proteinuria and prevent the progression of FSGS. Our results contribute to the increasing evidence of the role of mTOR signaling in the pathogenic mechanisms of FSGS. We found that human podocytes incubated in the presence of rFSGS PE present a relocalization of the Ragulator complex, which is key in the regulation of mTORC1 activity, to lipid rafts. Altogether, these results point to a pathogenic mechanism that deserves further investigation. 

To conclude on the plasma findings, the differential proteins that we identified can be considered candidate markers of FSGS. However, there are no arguments in our study to suggest that they are specific to rFSGS. For this purpose, a larger study comparing recurrent with non-recurrent patients would be necessary, along with the choice of additional control groups and in vitro and in vivo tests to confirm any pathogenic effects on renal glomeruli. The analytical procedure that we describe represents a step forward in the direct analysis of the plasma proteome, with clinical-evaluation applications for many other pathologies.

### 3.2. Proteomes of Plasma-Treated Podocytes

Furthermore, the present work addressed the proximal signaling changes—comprising protein recruitment in lipid rafts and protein phosphorylation—undergone by human podocytes when they are placed in contact with blood plasma—plasma-exchange fluid—from rFSGS patients for a short period of time. Some of the original aspects of our work are as follows. First, given the fact that recurrence can be a very rapid event—hours from kidney graft—we sought very early events induced by plasma. Second, we chose as controls, for the first time, patients that had followed a similar therapeutic protocol to FSGS before kidney graft, eliminating potential artefacts in cells due to the effect of immunosuppressive drugs. Third, we chose subproteomics as an alternative approach to unveil early and proximal changes in cells. In fact, our results also included a total-cell-proteome analysis, which yielded almost no significant differences, which was expected after a short incubation. This underlines the advantage of choosing subproteomic approaches, such as, in the present case, both raftomics and phosphoproteomics. 

A general review of the results obtained suggests that two main blocks of cell functions are differentially altered when podocytes are incubated with either rFSGS or control plasma. The first corresponds to functions regulated by the mTOR pathway, while the second relates to the regulation of the cytoskeleton. As shown in Figure 5, mTOR-associated proteins were mainly revealed in the raftomic set, whereas cytoskeleton-related proteins were mostly identified in the phosphoproteomics experiment. In addition, five proteins related to mTOR signaling were differentially recruited in rafts.

Within the proteins upregulated in the rafts from the rFSGS–PE-treated cells, we identified four subunits of the mTORC1 complex, namely, LAMTOR 2, 3, 4, and 5. This was concomitant with a decrease in four mitochondrial proteins, all subunits of ATPase 5. The Ragulator complex is critical for mTORC1 lysosomal recruitment [67] and its subsequent activation [68]. The Ragulator consists of five subunits and acts as an adaptor and a GTP-exchange factor for small Rag GTPases. The function of this complex in podocytes has been studied through LAMTOR1 ablation in mice, resulting in the attenuation of the renal phenotype [69]. Surprisingly, LAMTOR1 is the only component of the Ragulator that we did not find to be increased in the rafts from the rFSGS-treated cells. Whether the whole or only a part of the Ragulator complex are recruited in podocyte rafts upon exposure to rFSGS plasma remains to be confirmed. Generally, our results suggest the dysregulation of mTORC1 activation in response to rFSGS-PE. 

The aberrant activation of mTORC1 results in negative feedback to PI3K activation [70] and, in podocytes, it leads to cell injury, mimicking a feature of diabetic nephropathy [71,72]. Conversely, mTORC1 invalidation leads to podocyte dysfunction and proteinuria through the disruption of the autophagic flux [73]. The administration of the mTORC1 inhibitor, rapamycin, to patients with glomerulopathies results in proteinuria [74]. Altogether, these results suggest that mTOR signaling is finely tuned to ensure podocyte function. The negative regulation of mTORC1 is driven by the TSC (tuberous sclerosis complex). The inhibition of TSC results in mTORC1 activation in response to multiple stimuli, such as growth factors, glucose, and oxidative stress [75]. To establish the actual impact of the Ragulator raft recruitment suggested by our results on mTORC1 activity, we assessed the S6-kinase-phosphorylation status as an effector of mTORC1 activation. Our results showed that the S6 kinase expression—both total and phosphorylated—decreased following the PE incubation of podocytes, and in similar way in the cases of the rFSGS and control PE. Consequently, we cannot establish a link between Ragulator raft recruitment and decreases in mTORC1 activity. Further investigation is necessary.

If LAMTOR subunits are the most relevant proteins upregulated in rafts in the presence of rFSGS plasma, their counterparts (which were decreased in the rFSGS-treated and increased in the control-treated rafts) are four ATP5 ATP synthase (complex V) subunits involved in mitochondrial electron transport, along with sequestosome/p62; the latter is a central actor in the regulation of autophagy. Recent studies have shown that mTOR signaling controls mitochondrial dynamics [76,77], and thereby participates in the progression of mitochondrial myopathy [78] and cancer [79]. Complexes of ATP synthase might not necessarily be restricted to mitochondria, since they have been associated with detergent-resistant raft membranes [80,81,82,83,84,85]. The raft localization of these complexes has been reported as either mitochondrial [85] or ectopic [82]; the latter is associated with diverse functions. Therefore, our results may be indicative of an impairment in ATP-synthase functions in rFSGS–PE-treated podocytes compared to control–PE-treated cells.

The different phosphoproteins associated with mTORC1 include EIF4ENIF1, LARP1, SLC4A2 (upregulated in rFSGS–PE-incubated cells), BRD4, and NUFIP1 (downregulated). The EIF4ENIF1 and LARP1 phosphoproteins are involved in protein synthesis. In particular, EIF4ENIF1 (eIF4E nuclear import factor 1) is a shuttle protein ensuring the nuclear import of the eucaryotic-translation-initiation factor eIF4E, resulting in translation inhibition via interaction with importin 8 [86]. A specific study of protein translation in the conditions studied is necessary to clarify the role of mTORC1 in the cellular response to rFSGS plasma.

The phosphorylation of BRD4 and NUFIP1, along with the p62 decrease in rafts, suggest an impact on autophagy, one of the cellular functions targeted by mTOR signaling. The phosphorylation of BRD4 is characteristic of mitosis [87], but it has not been described in the context of autophagy. The inhibition of BRD4 is associated with autophagy activation [88,89,90]. The NUFIP1 is a receptor for the autophagic selective degradation of ribosomes in response to starvation [91] and to mechanical stress [92]. In both cases, no link has been established between phosphorylation and autophagy. Similarly, our study represents the first report of sequestosome-1/p62 with lipid rafts. The p62 is a scaffold protein involved in selective autophagy through interactions with polyubiquitinated proteins. The inhibition of the autophagic flow with increased p62 expression has been described in podocytes subjected to high-glucose conditions [93,94]. An increase in the autophagy flux has been reported in podocytes in response to high glucose, and it is protective against diabetes-induced glomerulosclerosis, which is characterized by mTOR activation [95]. Diabetic mice present high LC3B levels in podocytes. This contrasts with our observations of rFSGS–PE-incubated podocytes. Indeed, our results showed lower levels of LCRB at 30 min and 1 h of incubation, while the p62 levels were maintained compared to the control–PE-treated and non-treated cells. Altogether, we can speculate that rFSGS–PE induces a decrease in autophagy flow, and we hypothesize that the opposed presence of Ragulator and p62 in lipid rafts plays a role in this inhibition. These confirmation or rejection of these arguments requires further investigation.

Another striking, although not unexpected, observation arising from our results was the presence of changes in the phosphorylation status of a number of cytoskeletal proteins in the cells incubated with rFSGS–PE. However, the assessment of the cytoskeletal status for up to 24 h with phalloidin staining only showed modest differences between the cells treated with the two kinds of plasma. In all the cases, the podocytes seemed to maintain a fairly normal distribution of actin stress fibers. Although changes in cell morphology with increased cytoplasmic retraction were observed in the rFSGS–PE-treated cells, these alterations were far from representing complete disorganization. Therefore, observed differences at 30 min may be subtle early indicators of morphological changes that should be detected after longer exposure periods. Out of the eleven proteins linked with cytoskeletal function displaying significant differences in phosphopeptide abundance, six were upregulated in the rFSGS-treated and seven were upregulated in the control-treated cells, suggesting a unique phosphorylation pattern for each condition. Among the increased phosphopeptides in the rFSGS-treated cells, Hsp27 (HspB1) phosphorylation has been extensively studied with regards to the regulation of actin polymerization, cytoskeleton structure, and cell morphology, particularly in podocytes [96]. It is acknowledged that actin polymerization is a phenomenon associated with decreased cell motility and a less migratory phenotype. In podocytes, a migratory phenotype is a characteristic of a disorganized filtration barrier. Two phosphopeptides of Hsp27 were detected at 30 min of incubation, Ser82 and Ser65. The most studied to date, Ser82, was unchanged between the two studied conditions, while Ser65 was significantly increased in the rFSGS treatment. We confirmed through Western blotting that the Ser82 was unchanged after 30 min, but, conversely, it was significantly increased in the rFSGS- = treatment after 4 h of incubation, which indicates decreased actin polymerization and increased cell motility. This was in line with seminal studies on Hsp27’s role in actin polymerization in podocytes. The phosphorylation of Hsp27 in this residue was found to be increased in an in vivo puromycin model of glomerulopathy [97], and it was correlated with its ability to inhibit actin polymerization [98,99]. However, while Ser15, 78, and 82 have been extensively studied, little is known about the phosphorylation of Ser65, which, according, to our observations, was exclusive of rFSGS-treated cells at the early stage. 

Plectin (PLEC) phosphopeptides were also found to be upregulated in the rFSGS-treated cells. Plectin is a key actor in cytoskeletal organization, as a linker between microfilaments, intermediate filaments, and microtubules. It is suspected that PLEC and HSP27 interact with each other in human cells, as their homologous proteins in other species appear to interact in datasets [100]. However, PLEC has been scarcely studied in podocytes. Its expression is repressed in the adriamycin model of nephropathy, and siRNA silencing results in podocyte injury via the increased phosphorylation of integrin a6b4, FAK, and p38 MAPK [101]. In our study, no changes were observed between the two experimental conditions in the total PLEC expression of the podocytes. Conversely, we found a differential phosphorylation of S4396. This phosphosite has only been reported in the phagosomes of IFN-g-activated macrophages [102], while the close C-terminus phospho-S4642 decreases the ability of PLEC to interact with intermediate filaments [103].

Among the rest of the phosphoproteins augmented by the rFSGS plasma, MPRIP (p116RIP) is a myosin phosphatase and RhoA-GTPase-interacting protein. While its silencing leads to a loss of stress-fiber-associated RhoA, suggesting it acts as a scaffold linking RhoA to regulate myosin phosphatase at the stress fiber [104], its phosphorylation at S891 has been found in global phosphoproteomic studies in the context of mitosis [105,106] and cancer [107]. Furthermore, ORP3 (OSBPL3) is also linked with GTPases, as it interacts with R-Ras, which regulates cell adhesion and migration [108]. It is a protein kinase linking phosphoinositide metabolism with cytoskeleton dynamics and endocytosis, and it is phosphorylated in response to the loss of cell–cell contact. Phosphorylation has been found to lead to interaction with VAPA, inducing R-RAS activation, Akt phosphorylation, and β1-integrin activity [109]. This involves, among others, the S304 site that we found in the current study. This seems to be contradictory, as integrin-b1 activation is the result of nephrin signaling [110], and its expression in podocytes is essential for glomerular integrity [111]. In the present case, ORP3 phosphorylation may be understood as a response of the cell to the receptor-mediated sensing of cell-adhesion loss.

An unexpected finding was the higher level of phosphorylated AKAP12 in the podocytes treated with the rFSGS plasma. The AKAP12, a PKA-anchoring protein, was described as a marker of early FSGS lesions in glomerular parietal epithelial cells (PEC), but its expression in podocytes was not detected in the same work [112]. Therefore, we report, for the first time, the expression of AKAP12 by podocytes and a potential link between its phosphorylated form and an early response to rFSGS plasma. 

On the other hand, some phosphoproteins appeared to be comparatively decreased in the cells treated with the rFSGS–PE. Among them, the cytoplasmic adaptor NCK2 is of particular interest, as a known partner of nephrin and a regulator of actin assembly. The invalidation of this gene in podocytes results in decreased podocyte migration, without affecting the proliferation or expression of podocyte markers [113]. Decreases in the interactions between NCK2 and nephrin are among the effects observed in podocytes in response to angiotensin II stimulation, resulting in glomerular permeability and proteinuria [114]. No implications of NCK2 phosphorylation in podocyte biology have been reported to date. 

In addition to these cytoskeleton-related phosphoproteins, our results showed an increased presence in the rafts from the rFSGS-plasma-treated cells of LRRC16A, which belongs to the CARMIL family and is an inhibitor of the actin-capping protein, which interferes with actin polymerization and results in cytoskeleton disruption [35,113]. No associations between this protein and lipid rafts have been reported to date. Interestingly, only one protein, LARP1, is associated with both cytoskeleton regulation and mTORC1-associated signaling; a link between the two main differential pathways was therefore identified. 

Overall, our results must be taken with a high degree of caution due to the limited number of individuals studied. A critical point is the choice of controls. We chose kidney-transplanted patients undergoing humoral rejection as these patients receive treatments that are very similar to those administered to those with rFSGS. Nevertheless, slight differences in treatments may have an impact on the proteomes of incubated podocytes. Another major limitation of our study is the difference between its setup and that of the in vivo situation, in that our model of plasma incubation does not reproduce the glomerular filtration barrier, in which the contact of podocyte membranes with plasma proteins is limited by the presence of the glomerular basement membrane. Our setup could represent a second effect of circulating proteins on the glomerulus, with the first leading to the fragilization of the membrane. Generally, the observed specific changes in the raftome and the phosphoproteome in response to rFSGS PE point to the dysregulation of mTORC1, autophagy, and cytoskeletal organization. Subsequent targeted studies should (i) clarify whether these events are associated, (ii) establish their temporal sequence, (iii) confirm their in vivo relevance, and (iv) confirm whether any of these changes are specific to rFSGS plasma. 

Finally, aiming to cross-evaluate our plasma proteomics and our podocyte-proteomics data, we established the correlations between the levels of abundance of the proteins in both biological materials, in all the samples, and regardless of the PE origin (rFSGS or control). However, it must be noted that PE proteomics may not exactly reflect the proteins that are present in PE-incubation experiments, since some low-abundance proteins may be removed, along with the 12 most abundant proteins, during immunodepletion. With this point in mind, our analysis resulted in strong correlations between several plasma proteins and with protein changes in the podocytes (e.g., SIGLEC14/5, Multimerin 2, Apolipoprotein F, myeloperoxidase), and vice versa, with podocyte proteins highly affected by PE incubations (e.g., MBD3, FAM21A/C, and TWISTNB). Further investigation should determine whether there is a cause–effect relationship between the differential proteins in plasma that are strongly correlated with differential proteins in podocytes. Generally, future studies should determine whether any of these proteins constitute valuable in vitro markers and/or pathogenic factors. 

## 4. Materials and Methods

### 4.1. Patients and Controls

This project involved the analysis of blood-plasma samples from a retrospective cohort of patients with idiopathic nephrotic syndrome and healthy controls. These samples are currently available at the Henri Mondor Biobank (Créteil, France, NCT04174066). The contents and access to this biobank are regulated by ethical committee approval, CPP 2019-A00854-53. Healthy volunteers and nephropathic patients provided informed consent. The study enrolled five healthy individuals with no clinical history of kidney disease, four patients with post-transplant recurrent FSGS (rFSGS), and four patients with other nephropathies, used as patient controls (Table 1). Plasma (2 mL) was obtained from peripheral blood by centrifugation twice at 1500 g for 10 min. Control patients (CTRL) experienced humoral rejection months or years after renal transplantation and consequently received several sessions of plasma exchange (PE). All patients were subjected to similar immunosuppressor therapies before or after plasma-exchange session. 

### 4.2. Plasma Collection and Preparation

First post-transplant PE fluid was collected immediately after plasmapheresis session and frozen at −80 °C. Plasma was obtained from healthy individuals through blood centrifugation. Protein content was evaluated by absorbance measurement at 595 nm using the Bradford reagent. The mean protein contents were not significantly different between control and rFSGS samples (61.6 ± 10.1 g/L vs. 94.6 ± 42.3 g/L respectively).

#### 4.2.1. EV Preparation

Extracellular vesicles were obtained by a centrifugation method based on the protocol published by Geiger’s laboratory [115]. All the steps were carried out at 4 °C. Briefly, plasma was centrifuged at 3300× *g* for 20 min to remove platelets. Supernatants were collected in clean tubes and diluted 2-fold with ice-cold PBS and centrifuged at 20,000× *g* for 1 h. Pelleted extracellular vesicles were then washed twice with the same volume of ice-cold PBS and suspended in 5% SDS, 50 mM triethylammonium bicarbonate (TEAB).

#### 4.2.2. Plasma Immunodepletion Using Proteome PurifyTM Kit

Proteome PurifyTM 12 Human Serum Protein Immunodepletion Resin kit (R&D Systems, Minneapolis, MN, USA) was used to deplete the 12 most abundant proteins according to the manufacturer’s protocol, with some modifications. Resin (1 mL) was centrifuged for 30 s at 1000× *g*. The PE and plasma were thawed on ice, and 10 µL of each sample was mixed with 500 µL of PBS and incubated with the resin under rotary agitation for 1 h at room temperature. After incubation, samples were centrifuged on Spin-X 0.22 µm cellulose-acetate-membrane filters (Corning Costar^®^, Corning, NY, USA) to remove the resin and further centrifuged on 3 kDa Microcon (Millipore, Bedford, MA, USA) to concentrate the depleted samples.

### 4.3. Suspension Trapping (S-Trap)

The S-Trap^TM^ micro-spin column digestion was performed on OptiPrep^TM^ raft fractions according to the manufacturer’s protocol. Briefly, proteins were precipitated overnight using 10% TCA final concentration and washed four times with cold ethanol. Proteins were resuspended and solubilized in 5% SDS and 50 mM TEAB (pH 7.55), reduced with 20 mM tris(2-carboxyethyl)phosphine (TCEP) solution, and alkylated by the addition of chloroacetamide to a final concentration of 50 mM. Aqueous phosphoric acid was added to a final concentration of 1.2%. Colloidal protein particulate was formed with the addition of 231 µL of S-Trap binding buffer (90% aqueous methanol, 100 mM TEAB, pH7.1). The mixture was placed on S-Trap micro 1.7 mL columns and centrifuged at 4000× *g* for 10 s. Columns were washed five times with 150 µL S-Trap binding buffer and centrifuged at 4000× *g* for 10 s with 180-degree rotation of the columns between washes. Samples were digested with 2 µg of trypsin (Promega) at 47 °C for 1 h. Peptides were eluted with 40 µL of 50 mM TEAB, followed by 40 µL of 0.2% aqueous formic acid and by 35 µL 50% acetonitrile containing 0.2% formic acid. Peptides were finally vacuum dried. Samples were resuspended in 20 µL of 1% ACN and 0.1% TFA in HPLC-grade water.

### 4.4. High pH Fractionation

Samples were fractionated by in-tip high-pH reverse-phase chromatography, using 1 mg of C18-AQ 5 µm beads (Dr. Maisch, GmbH, Ammerbuch, Germany) per sample. Beads were conditioned twice with 50 µL of 50% ACN and twice with 50 µL of 0.1% TFA, and centrifuged 2 min at 1500× *g*. Peptides were resuspended in 50 µL of 0.1% TFA and added onto the stage tip centrifuged 2 min at 1500× *g*. The stage tip was washed with 50 µL of HPLC-grade water and the peptides were sequentially eluted with 0.1% trimethylamine and increasing percentages of ACN (5%, 7.5%, 10%, 12.5%, 15%, 17.5%, 20%, and 50%). The 26 fractions were concatenated in 13 fractions (F1-F14, F2-F15, F3-F16 (…) F12-F25, F13-F26). These 13 fractions were dried and resuspended in 14 µL of 10% ACN, 0.1% TFA in HPLC-grade water.

### 4.5. Cell Culture and Incubations

Immortalized human podocytes (AB 8/13) were cultured in collagen-A-coated plates (0.1 mg/mL) in RPMI 1640 medium containing 10% fetal calf serum (FCS), 1% penicillin, 1% streptomycin, and 1% insulin–transferrin–selenium at 33 °C. Differentiation was induced by maintaining stable podocyte cell lines at 37 °C for 10 days. Before treatment, podocytes were starved in 2% FCS for 4 h. Podocytes were then treated with 10% plasma exchange from 8 different patients (4 controls and 4 rFSGS, Table 1) for 30 min. After treatment, cells were washed thoroughly three times with ice-cold PBS. For lipid-raft analysis, cells were directly prepared without freezing the cell pellet. For the phosphoproteome, cells were lysed for 1 h at 4 °C under rotary agitation with RIPA 1× with 1% SDS as final concentration, with PhosSTOP^TM^, complete protease-inhibitor cocktail (Roche), 1 µL of MgCl2 100 mM, and 1 µL benzonase and stored at −80 °C before analysis. In a separate series of experiments, podocytes were incubated with 10% plasma-exchange fluid for 30 min, 1 h, 4 h, and 24 h. Cell-protein extracts were prepared in lysis buffer B (150 mM NaCl, 10 mM Tris HCl pH 7.5, 2 mM DTT, 10% glycerol, 1 mM EDTA, 1% NP40, 1 mM protease inhibitors, 1 mM NaF, and 1 mM sodium orthovanadate).

### 4.6. Lipid-Raft Preparation 

Lipid-raft-like microdomains were obtained by using a detergent-free method based on that described by McDonald and Pike (32). Podocytes were resuspended in 800 μL of MBS/Na_2_CO_3_ buffer (25 mM MES, 150 mM NaCl, 250 mM Na_2_CO_3_, pH6; supplemented with 1 mM PMSF and phosphatase and protease-inhibitor cocktails) and lysed by passaging 20 times through a 21G needle, followed by sonication 3 times for 60 s in a Vibra Cell 75022 sonicator. The homogenate was mixed with two volumes of 60% OptiPrep^TM^ (Axis Shield, Dundee, UK) for a final volume of 2 mL of 40% OptiPrep^TM^. A three-step discontinuous density gradient was made by sequentially placing 2 mL of either 30% OptiPrep^TM^ in MBS/Na_2_CO_3_ buffer and 1 mL of 5% OptiPrep^TM^. The mixture was spun in a TL-100 rotor at 268,000× *g* for 2 h in an Optima MAX-XP ultracentrifuge (Beckman Coulter, Brea, CA, USA). After spinning, one fraction of 600 µL and, subsequently, 5 fractions of 900 µL were collected from top to bottom. After verification, fraction 2 containing rafts was subjected to subsequent analysis. To analyze the distribution of flotillin-1, fractions were precipitated by addition of 10% trichloroacetic acid (final concentration), incubated overnight at −20 °C, and washed three times in cold ethanol. The resulting dry protein pellets were solubilized in equal volumes of 1× Laemmli buffer and analyzed by Western blot.

### 4.7. Filter-Aided Sample Preparation (FASP) 

The FASP was performed on 200 µg of proteins according to [116] to digest proteins for the phosphoproteome. Briefly, samples were reduced with 0.1 M dithiotreitol (DTT) at 60 °C for 1 h. Proteins were transferred to Microcon filter units (30 kDa cut-off) and washed twice with 200 μL of UA buffer (0.1 M Tris, 8 M urea, pH 8.9) and concentrated by centrifugation at 14,000× *g* for 15 min. Proteins were alkylated with 100 μL of IAA buffer (0.05 M iodoacetamide, 0.1 M Tris, pH 8.9) at room temperature in the dark for 20 min and centrifuged at 14,000× *g* for 10 min. Proteins were then washed twice by adding 100 μL of UA buffer before centrifugation at 14,000× *g* for 10 min, and twice with 100 μL of ABC buffer (0.05 M NH_4_HCO_3_) before centrifugation at 14,000× *g* for 10 min. Filter units were transferred to new collection tubes and samples were incubated with 40 μL of ABC buffer containing 1.6 μg of trypsin in a humidity chamber at 37 °C for 18 h. Tubes were centrifuged at 14,000× *g* for 10 min, 40 μL of ABC buffer was added, and tubes were centrifuged again. Peptides were finally recovered in collection tubes.

### 4.8. Phosphopeptide Enrichment

Phosphopeptide enrichment was carried out with titanium dioxide (TiO_2_) and phosphopeptide purification was performed with graphite carbon (GC). Samples were clarified by centrifugation at 14,000× *g* for 10 min. Protein concentration was determined with DC assay (Biorad) on clarified lysates. In total, 210 µg of protein was digested by FASP (34). After digestion, 20 µg of protein was saved for total proteome analysis, while 190 µg was used for subsequent phophopeptide enrichment. Phosphopeptide enrichment was carried out using a Titansphere TiO_2_ spin tip (3 mg/200 μL, Titansphere PHOS-TiO, GL Sciences Inc., Tokyo, Japan), with an estimated 1.2 mg of digested proteins for each biological replicate. Briefly, the TiO2 spin tips were conditioned with 20 µL of solution A (80% acetonitrile, 0,1% TFA), centrifuged at 3000× *g* for 2 min, and equilibrated with 20 µL of solution B (75% acetonitrile, 0.075% TFA, 25% lactic acid), followed by centrifugation at 3000× *g* for 2 min. Peptides were resuspended in 10 µL of 2% TFA, mixed with 100 µL of solution B, and centrifuged at 1000× *g* for 10 min. Sample was applied back to the TiO_2_ spin tips two more times in order to increase the adsorption of the phosphopeptides to the TiO_2_. Spin tips were washed, sequentially, with 20 µL of solution B and twice with 20 µL of solution A. Phosphopeptides were eluted by the sequential addition of 50 µL of 5% NH_4_OH and 50 µL of 5% pyrrolidine. Centrifugation was carried out at 1000× *g* for 5 min.

Phosphopeptides were further purified using GC spin tips (GL-Tip, Titansphere). Briefly, the GC spin tips were conditioned with 20 µL of solution A, centrifuged at 3000× *g* for 2 min, and equilibrated with 20 µL of solution C (0.1% TFA in HPLC-grade water), followed by centrifugation at 3000× *g* for 2 min. Eluted phosphopeptides from the TiO_2_ spin tips were added to the GC spin tips and centrifuged at 1000× *g* for 5 min. The GC spin tips were washed with 20 µL of solution C. Phosphopeptides were eluted with 70 µL of solution A (1000× *g* for 3 min) and vacuum-dried.

### 4.9. Nano-LC-MS/MS-Protein Identification and Quantification

Samples were resuspended in 35 µL of 1% ACN, 0.1% TFA in HPLC-grade water. For each run, 5 µL was injected in a nanoRSLC-Q Exactive PLUS (RSLC Ultimate 3000) (Thermo Scientific, Waltham, MA, USA). Peptides were loaded onto a µ-precolumn (Acclaim PepMap 100 C18, cartridge, 300 µm i.d. × 5 mm, 5 µm) (Thermo Scientific) and separated on a 50 cm reversed-phase liquid-chromatography column (0.075 mm ID, Acclaim PepMap 100, C18, 2 µm) (Thermo Scientific). Chromatography solvents were (A) 0.1% formic acid in water, and (B) 80% acetonitrile, 0.08% formic acid. Peptides were eluted from the column with the following gradients: 5% to 40% B (120 min), 40% to 80% (5 min). At 125 min, the gradient returned to 5% to re-equilibrate the column for 20 min before the next injection. Two blanks were run between samples to prevent sample carryover. Peptides eluting from the column were analyzed by data-dependent MS/MS, using a top-10 acquisition method. Peptides were fragmented using higher-energy collisional dissociation (HCD). Briefly, the instrument settings were as follows: resolution was set to 70,000 for MS scans and 17,500 for the data-dependent MS/MS scans in order to increase speed. The MS AGC target was set to 3 × 10^6^ counts, with maximum injection time set to 200 ms, while MS/MS AGC target was set to 1 × 10^5^, with maximum injection time set to 120 ms. The MS-scan range was from 400 *m*/*z* to 2000 *m*/*z*. Duration of dynamic exclusion was set to 30 s. Three separate mass-spectrometry runs (i.e., technical replicates) were acquired for each biological replicate under the identical mass spectrometric conditions to account for instrument-related variability and to improve accuracy of the label-free quantification.

### 4.10. MS-Data Processing and Bioinformatic Analysis

The MS-data processing and bioinformatics were performed as previously described, with some modifications [117]. Briefly, raw MS files were processed with the MaxQuant software version 1.5.8.3 and searched with the Andromeda search engine against the human UniProt database (release May 2019, 20,199 entries). To search for parent mass and fragment ions, we set the mass deviation at 4.5 ppm and 20 ppm, respectively. The minimum peptide length was set to seven amino acids and strict specificity for trypsin cleavage was required, allowing up to two missed cleavage sites. Match between runs was allowed. Carbamidomethylation (Cys) was set as fixed modification, whereas oxidation (Met) and protein N-terminal acetylation phosphorylation (Ser, Thr, Tyr) were set as variable modifications (for phosphoproteomics analysis only). The false discovery rates (FDRs) at the protein and peptide level were set to 1%. Scores were calculated in MaxQuant, as described previously [117].

Statistical and bioinformatic analyses, including heatmaps, were performed with Perseus software (version 1.5.5.3), freely available at www.perseus-framework.org (accessed on 28 September 2020). Gene Ontology (GO) annotation was performed on Perseus software. We retrieved annotations for the GO cellular-component terms “membrane”, “extracellular vesicular exosome”, “plasma membrane”, “mitochondrion”, and “endoplasmic reticulum membrane”.

The phosphopeptide-output table and the corresponding logarithmic intensities were used for phosphopeptide analysis. The phosphopeptide table was expanded to separate individual phosphosites, and we kept all sites identified in four replicates in at least one group (CTRL vs. rFSGS). Missing values were imputed using width = 0.2 and down-shift = 3. We represented on a heatmap the significantly altered phosphosites (*t*-test S0 = 0.1, FDR = 0.05).

The protein-groups output table was used for total proteome analysis. We kept only proteins identified in all four replicates in at least one group (CTRL vs. rFSGS). Missing values were imputed using width = 0.3 and down-shift = 2.5. For volcano plot, we used *t*-test, S0 = 0.5 and FDR = 0.01 (for class B proteins), and the outer volcano used S0 = 0.5, FDR = 0.001 (class-A proteins). 

### 4.11. Immunodetection and Fluorescence Microscopy

Selected markers were validated by Western blot analysis. Protein extracts (35 µg) were subjected to SDS-page electrophoresis on 10% polyacrylamide gels. Primary antibodies targeted human Flotillin-1 (mouse monoclonal, BD Biosciences, 1:1000), LAMTOR2 (rabbit polyclonal, Invitrogen, 1:1000), p62-sequestosome 1 (rabbit polyclonal, Proteintech, 1:1000), phosphorylated S6 kinase (mouse monoclonal, Santa Cruz, 1:1000), total S6 kinase (mouse monoclonal, Santa Cruz, 1:1000), LC3B (rabbit polyclonal, Cell Signaling, 1:1000), phosphorylated Hsp27 (Ser82, rabbit monoclonal, Abcam, 1:1000), total Hsp27 (rabbit monoclonal, Abcam, 1:1000), alpha-tubulin (rat monoclonal, Abcam, 1:2000), and GAPDH (rabbit polyclonal, Cell Signaling, 1:3000). The HRP-coupled secondary antibodies were used at 1:3000 (anti-rabbit and anti-mouse) and 1:2500 (anti-rat) dilutions. Chemiluminescence detection and band quantitation were performed with a Vilber Lourmat camera and associated software. Band intensities were statistically analyzed by the ratio of paired *T*-test with the Graph Pad 8 software package. Statistical significance was attributed to *p* < 0.05 values. Cytoskeletal F-actin fibers were visualized by incubation with FITC-conjugated phalloidin (Molecular Probes, Eugene, OR, USA). The slides were covered with Vectashield mounting medium containing DAPI, and viewed with a fluorescence microscope (Zeiss, Germany) using blue and green filters.

## Figures and Tables

**Table 1:** Demographic and clinical characteristics of patients involved in the study.

Group	Sex (M/F)	Age	Diagnosis	Prot/Creat (mg/mmol)	Number of Transplant	Biopsy Result	Treatments
Ctrl1	M	74	Other nephropathy	32.67	2nd	HR	CS/MMF/CNI/PE/RTX/Ig
Ctrl2	M	60	Other nephropathy	37.93	1st	HR	CS/MMF/mTORi/CNI /PE/RTX/Ig
Ctrl3	M	62	IgA nephropathy	91.49	1st	HR	CS/MMF/CNI/PE/RTX/Ig
Ctrl4	F	45	Diabetic nephropathy	67.1	1st	HR	CS/MMF/mTORi/PE/RTX/Ig
rFSGS1	M	31	FSGS	229.59	2nd	rFSGS	CS/MMF/CNI/PE/RTX/Ig
rFSGS2	M	21	FSGS	436	1st	rFSGS	CS/CNI/PE/RTX/Ig
rFSGS3	F	70	FSGS	129.34	1st	rFSGS	CS/PE/RTX/Ig
rFSGS4	F	48	FSGS	800	2nd	rFSGS	CS/MMF/CNI/PE/RTX/Ig

HR: humoral rejection; CS: corticosteroids; MMF: mycophenolate mofetil; mTORi (everolimus); CNI: calcineurin inhibitor (cyclosporin or tacrolimus); PE: plasma exchange; RTX: rituximab; Ig: immunoglobulin.; Ritux: rituximab; Cyclo: cyclosporin.

## Data Availability

The mass spectrometry proteomics data have been deposited to the ProteomeXchange Consortium via the PRIDE partner repository with the dataset identifier PXD042379.

## Supplementary Materials

The supporting information can be downloaded at: https://www.mdpi.com/article/10.3390/ijms241512124/s1.
