# Supplementary material for: Proteomics of Plasma and Plasma-Treated Podocytes: Application to Focal and Segmental Glomerulosclerosis
Source: Int J Mol Sci. 2023 Jul 28;24(15):12124. doi: 10.3390/ijms241512124 (PMC10418338; doi:10.3390/ijms241512124)
Supplement: Supplementary file 1 [file ijms-24-12124-s001.zip › figures_supplemental-revised version.pdf]

Figure S1

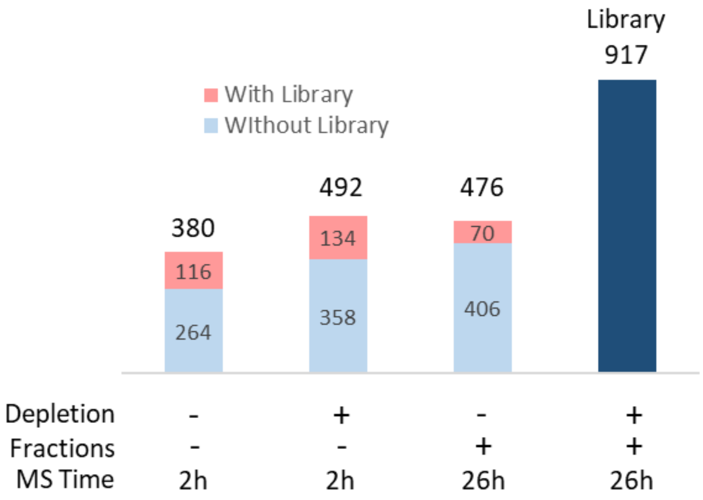

Figure S1. Building of a spectral library with TOP12 depletion and extensive fractionation. For comparison, samples were also depleted or non-depleted with or without fractionation. High-pH fractionation was performed on plasma exchange, 26 fractions were collected and pooled in 13 fractions. Each fraction was run in a 2-h gradient. Number of proteins for each analysis was reported in histograms (in light blue). Additional proteins (in pink) identified with the library were also indicated.

Figure S2

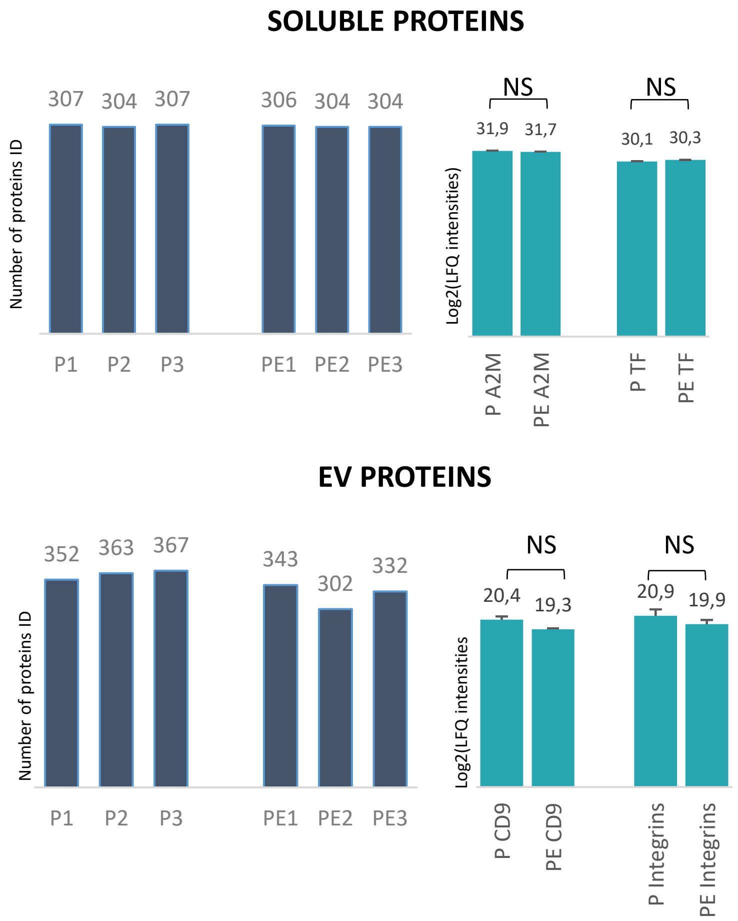

Figure S2. Similarity between plasma and PE from the same individual. Difference between TOP12 plasma and PE from same individual. For soluble and EV plasma proteins, number of identified proteins were reported. Comparison of log2 LFQ intensities of plasma proteins (A2M and TF) and protein markers of EV (CD9 and integrins).

Figure S3

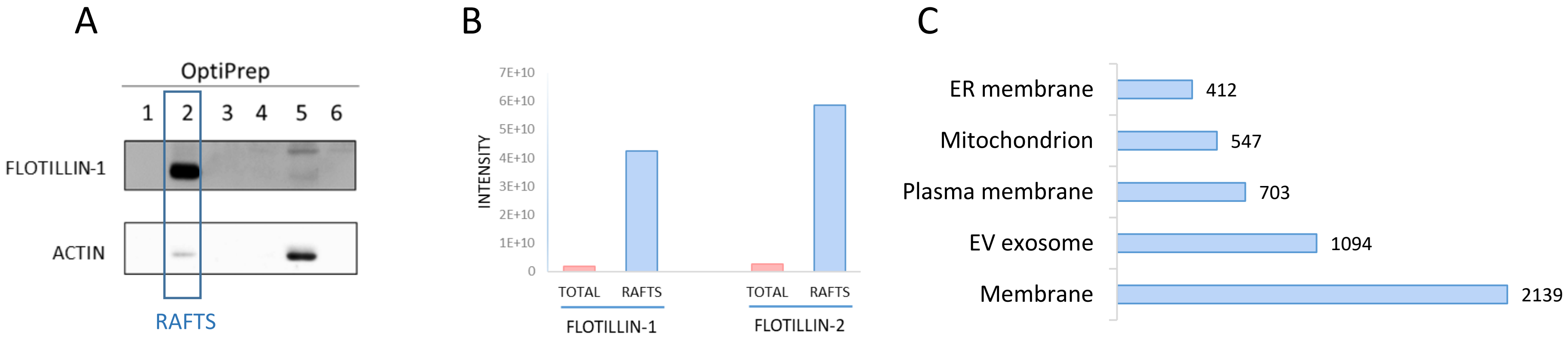

Figure S3. Raft purity assessment. (A) Detection of lipid raft marker, flotillin-1 and non-lipid raft marker, actin, in fractions obtained from immortalized human podocytes treated with control and rFSGS PE. Cells were lysed and subjected to Optiprep density gradient. Proteins were precipitated with 10% TCA final concentration and resuspended in Laemmli buffer 1X for WB. (B) Intensities calculated by Maxquant for flotillin-1 and flotillin-2. Proteins were digested by S-Trap and analysed by LC-MS/MS. Intensities were calculated as the sum of all the intensities of the two proteins across all samples. (C) Gene Ontology Analysis of the 3163 proteins identified in raft analysis. Histograms indicate the number of proteins annotated with “membrane”, “plasma membrane”, “ER membrane” (for Endoplasmic Reticulum membrane), “Mitochondrion”, “EV exosome” (for Extracellular Vesicular Exosome) terms.

Figure S4

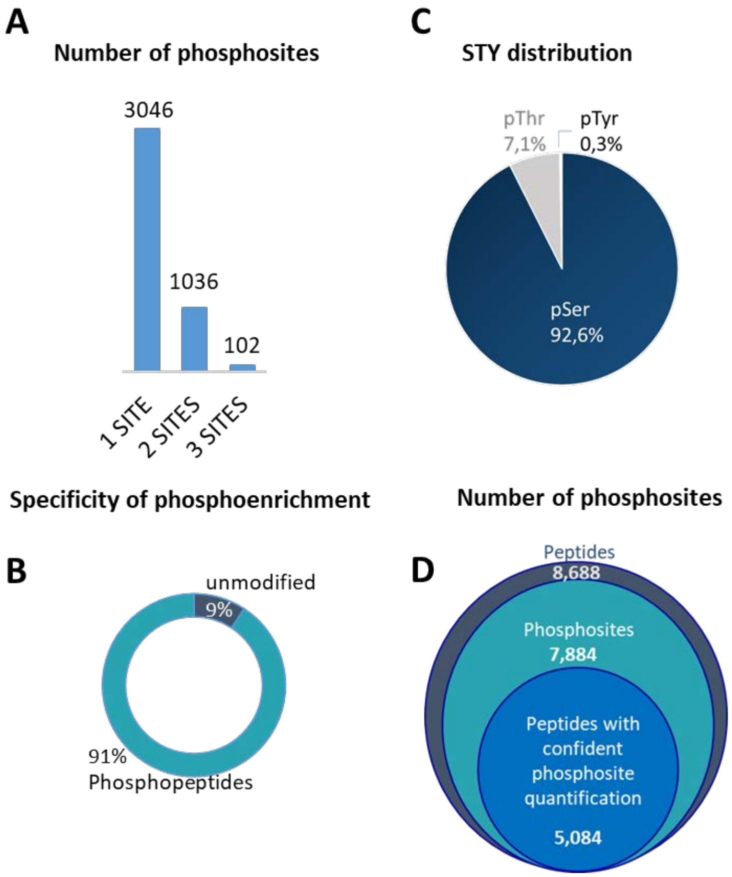

Figure S4. Synthesis of phosphoproteomic results. (A) Number of singly, doubly and triply phosphorylated peptides. (B) Specificity of phosphoenrichment. Donut chart indicating the percentage of phosphorylated (91%) and unmodified peptides (9%). (C) Pie chart representing the distribution of phosphorylation sites on serine (92,6%), threonine (7,1%) and tyrosine sites (0,3%). (D) Venn diagram showing the number of peptides, phosphosites and peptides with confident phosphosite quantification (with localization probability>0.75, at least 4 values for each group).
